# Low awareness of the transitivity assumption in complex networks of interventions: a systematic survey from 721 network meta-analyses

**DOI:** 10.1186/s12916-024-03322-1

**Published:** 2024-03-13

**Authors:** Loukia M. Spineli, Chrysostomos Kalyvas, Juan Jose Yepes-Nuñez, Andrés Mauricio García-Sierra, Diana C. Rivera-Pinzón, Svenja E. Seide, Katerina Papadimitropoulou

**Affiliations:** 1 Midwifery Research and Education Unit (OE 9210), Hannover Medical School, Carl-Neuberg-Straße 1, 30625 Hannover, Germany; 2 Biostatistics and Research Decision Sciences, MSD Europe Inc., Brussels, Belgium; 3 School of Medicine, Universidad de los Andes, Bogotá, Colombia; 4 Pulmonology Service, Internal Medicine Section, Fundación Santa Fe de Bogotá University Hospital, Bogotá, Colombia; 5 School of Global Health Management and Informatics, University of Central Florida, Orlando, USA; 6 Institute of Medical Biometry, University Hospital Heidelberg, Heidelberg, Germany; 7 Health Economics and Market Access, Amaris Consulting, Lyon, France

**Keywords:** Network meta-analysis, Transitivity assumption, Consistency assumption, Systematic review, Empirical study

## Abstract

**Background:**

The transitivity assumption is the cornerstone of network meta-analysis (NMA). Violating transitivity compromises the credibility of the indirect estimates and, by extent, the estimated treatment effects of the comparisons in the network. The present study offers comprehensive empirical evidence on the completeness of reporting and evaluating transitivity in systematic reviews with multiple interventions.

**Methods:**

We screened the datasets of two previous empirical studies, resulting in 361 systematic reviews with NMA published between January 2011 and April 2015. We updated our evidence base with an additional 360 systematic reviews with NMA published between 2016 and 2021, employing a pragmatic approach. We devised assessment criteria for reporting and evaluating transitivity using relevant methodological literature and compared their reporting frequency before and after the PRISMA-NMA statement.

**Results:**

Systematic reviews published after PRISMA-NMA were more likely to provide a protocol (odds ratio (OR): 3.94, 95% CI: 2.79–5.64), pre-plan the transitivity evaluation (OR: 3.01, 95% CI: 1.54–6.23), and report the evaluation and results (OR: 2.10, 95% CI: 1.55–2.86) than those before PRISMA-NMA. However, systematic reviews after PRISMA-NMA were less likely to define transitivity (OR: 0.57, 95% CI: 0.42–0.79) and discuss the implications of transitivity (OR: 0.48, 95% CI: 0.27–0.85) than those published before PRISMA-NMA. Most systematic reviews evaluated transitivity statistically than conceptually (40% versus 12% before PRISMA-NMA, and 54% versus 11% after PRISMA-NMA), with consistency evaluation being the most preferred (34% before versus 47% after PRISMA-NMA). One in five reviews inferred the plausibility of the transitivity (22% before versus 18% after PRISMA-NMA), followed by 11% of reviews that found it difficult to judge transitivity due to insufficient data. In justifying their conclusions, reviews considered mostly the comparability of the trials (24% before versus 30% after PRISMA-NMA), followed by the consistency evaluation (23% before versus 16% after PRISMA-NMA).

**Conclusions:**

Overall, there has been a slight improvement in reporting and evaluating transitivity since releasing PRISMA-NMA, particularly in items related to the systematic review report. Nevertheless, there has been limited attention to pre-planning the transitivity evaluation and low awareness of the conceptual evaluation methods that align with the nature of the assumption.

**Supplementary Information:**

The online version contains supplementary material available at 10.1186/s12916-024-03322-1.

## Background

Systematic reviews have long been advocated for providing the best evidence to inform decision-making in various health fields, provided they have been rigorously planned, conducted, and reported. The explosive rate at which systematic reviews are being published attests to the popularity of this research tool within the broad medical community [1]. A pairwise meta-analysis, following a systematic review, is the simplest form of evidence synthesis, comparing only two interventions for a specific health condition and population. When the research question includes more than two interventions, the pairwise meta-analysis is extended to incorporate trials investigating different interventions. The corresponding model is known as network meta-analysis (NMA).

The methodological advances in NMA and the number of published systematic reviews with multiple interventions have flourished exponentially over the last decade [2–4]. Considering the increasing number of alternative intervention options and the limited available resources to demonstrate their benefit-harm balance via randomised controlled trials, NMA plays a crucial role in generating the best evidence for timely decision-making [5, 6]. However, the quality of the conclusions delivered to the end-users depends on the validity of the underlying assumptions that define this evidence synthesis tool.

Network meta-analysis, as an extension of the pairwise meta-analysis, has been developed based on the same assumptions: sufficient clinical and methodological similarity and statistical homogeneity. The clinical and methodological similarity, known as the transitivity assumption [5, 7], expands from the similarity within comparison to the similarity across comparisons [8]. The transitivity assumption, first arguably coined by Baker and Kramer [7], states that pre-specified clinical and methodological characteristics of the synthesised trials, acting as effect modifiers, are similarly distributed across the observed comparisons in the network. Interchangeably, transitivity further implies the following: (a) the interventions of the network are similar across the corresponding trials; (b) missing interventions in each trial of the network are missing at random; (c) observed and unobserved underlying treatment effects are exchangeable; and (d) participants could be jointly randomisable to any intervention in the network (Table 1) [5].

**Table 1:** Interchangeable interpretations of the transitivity assumption (Salanti [5])

**(a) Similar interventions in different trials**
The interventions of the network do not differ systematically across the corresponding trials. Namely, in a triangle network with interventions A, B, and C, intervention A would be similar in AB and AC trials. The same holds for intervention B which appears in BC and AB trials, and intervention C in AC and BC trials
**(b) Missing-at-random treatments**
Missing interventions in each trial of the network are missing for reasons unrelated to their benefit-harm profile. Namely, interventions A, B, and C are randomly missing in BC, AC, and AB trials
**(c) Exchangeable missing and observed relative treatment effects**
Underlying treatment effects of any observed and unobserved comparison do not differ beyond what is expected by the between-trial heterogeneity alone. For instance, the AB trials provide evidence for comparison AB only. Under the random-effects model, had these trials included intervention C, the underlying treatment effect of AC and BC comparisons could have been estimated, assuming that these missing treatment effects are exchangeable with the corresponding underlying treatment effects estimated directly in AC and BC trials, respectively
**(d) Jointly randomisable participants**
If all network interventions could be investigated in one trial, the participants would be eligible to be randomised to any intervention. Namely, the participants share a similar demographic and clinical profile that makes them suitable for any network intervention for their underlying condition
**(e) Similar treatment comparisons concerning important effect modifiers**
Different observed treatment comparisons comprise clusters of several trials. These clusters are considered to be similar regarding the distribution of important effect modifiers. Hence, if AB and AC trials are similar in terms of the distribution of important effect modifiers, the indirect estimate for BC using these two sets of trials will be valid

The statistical representation of transitivity is known as (statistical) consistency [5]. Unlike transitivity assessment, consistency requires a closed loop of at least three interventions. Transitivity ensures that indirect evidence (obtained from different sets of trials sharing one or more common comparators) validly describes the treatment effect of the corresponding unobserved treatment comparison. Consistency signifies agreement between direct and indirect evidence, ensuring a valid mixed (NMA) treatment effect. As an extension of transitivity, consistency can be formulated using the interchangeable interpretations presented in Table 1 [5]. While transitivity and consistency essentially represent the same assumption, they are typically investigated separately [5].

Transitivity is an untestable assumption and rests on clinical and epidemiological grounds [2]. Hence, content expertise, well-validated effect modifiers, and subjective judgements are required to determine its validity [9–11]. Evaluating transitivity involves the meticulous scrutiny of the included trials based on the five interchangeable interpretations (Table 1), which can be assessed conceptually [5, 9, 12]. Statistical methods can also be employed to investigate the comparability of treatment comparisons in terms of the distribution of effect modifiers (item (e) in Table 1), provided there are sufficient data. Network meta-regression may improve the plausibility of transitivity and mitigate confounding bias in the indirect estimates when there are enough trials to inform the comparisons, and the effect modifiers are comprehensively reported [5, 13]. However, effect modifiers are often underreported, with participant-specific characteristics being averaged over trial arms, and comparisons include a limited number of trials, complicating the conceptual and statistical evaluation of the transitivity assumption [14–16].

Establishing the plausibility of transitivity is vital because the benefits of randomisation do not generalise across randomised controlled trials included in the network. If there is substantial clinical and methodological dissimilarity in the evidence base, the feasibility of conducting NMA may be implausible [17]. Potential violation of the transitivity assumption compromises the validity of the indirect estimates and, consequently, the estimates derived from NMA for some or all possible comparisons in the network [18]. When transitivity is questionable, recommendations advocate resorting to meta-regression to obtain the adjusted indirect effects (provided there are enough data) [5, 19, 20], splitting the network to sub-networks where transitivity is justified (if applicable) [9], or refraining from performing NMA [5].

Most empirical studies on the evaluation and reporting quality of the underlying assumptions for NMA have primarily focused on the quality of the indirect comparisons [19, 21, 22]. Donegan et al. [12] conducted the first survey investigating the reporting quality of the transitivity assumption. The authors devised specific quality assessment criteria based on relevant literature recommendations. These criteria were applied to 43 published systematic reviews [12]. Eligible reviews were required to include at least one indirect comparison of two interventions obtained using the Bucher method [23] while excluding reviews that conducted NMA, thus, providing limited empirical evidence.

Since the study by Donegan et al. [12] and the advent of the PRISMA extension statement for NMA (PRISMA-NMA) [11], the reporting quality of the transitivity assumption has yet to be revisited empirically. Therefore, conducting a comprehensive survey in that direction is timely and imperative. The objective of the present systematic survey was to revisit the reporting and evaluation quality of the transitivity assumption by including a broader set of published systematic reviews with multiple interventions, irrespective of the network structure, to allow for an extensive and updated evidence base. With the premise also to identifying any improvements or gaps in the reporting quality of the transitivity assumption over time, we considered systematic reviews published before and after the release of PRISMA-NMA while expanding upon the quality assessment criteria of Donegan and colleagues [12].

The rest of the article is organised as follows: first, we outline the steps taken to conduct the systematic survey on published systematic reviews with NMA and describe the quality assessment criteria for reporting and evaluating the transitivity assumption. Then, we summarise the extracted information in textual, tabular, and graphical formats. The “Discussion” section presents the study’s findings, strengths, and limitations while highlighting deficiencies in reporting and evaluation of transitivity with suggestions for improvement and increased attention in future systematic reviews with multiple interventions. Finally, we conclude with recommendations for good reporting and evaluation practices for the transitivity assumption.

## Methods

In this systematic survey, we have used the term NMA to describe the synthesis of at least three trials comparing different sets of interventions without distinguishing between methods for anchored indirect comparisons and networks with closed loops of interventions [24]. The network size and structure may determine the methods for assessing transitivity. For example, star-shaped networks preclude the evaluation of consistency, and meta-regression is not feasible in a sparse network. However, the network features do not dictate the plausibility of transitivity, which must be adequately evaluated regardless [13].

### Systematic review selection

Initially, we considered the collection by Petropoulou et al. [3], which included 456 systematic reviews of multiple interventions published between 1999 and 2015. However, considering that Donegan et al. [12] published their survey in November 2010, we restricted our dataset to systematic reviews published from 2011 onwards. The rationale for this restriction was that subsequent systematic reviews may have incorporated the recommendations by Donegan et al. [12] in their reporting and assessment of the transitivity assumption, potentially improving the quality of their conclusions.

The collection from Petropoulou et al. [3] included systematic reviews with at least four interventions. In a previous empirical study [25], we updated their collection by including systematic reviews with three interventions, following the inclusion and exclusion criteria of Petropoulou and colleagues [3]. This two-stage approach led to a total of 361 eligible systematic reviews published between January 2011 and April 2015. Additional file 1: Table S1 presents the screening process of systematic reviews with at least three interventions using the collection of Petropoulou et al. [3], the database of NMAs accessed using the nmadb R package [26], and the previous empirical study [25].

The PRISMA-NMA statement was published in June 2015 [11]. We adopted a pragmatic approach to retrieve eligible systematic reviews published after 2015 with at least three interventions. To be consistent with the previous step, where we collected 361 systematic reviews published between January 2011 and April 2015, we opted to include a total of 360 systematic reviews. We aimed for 60 systematic reviews per year from 2016 to 2021 to ensure a broader timeframe and create an up-to-date evidence base. Then, we employed the search algorithm and the inclusion and exclusion criteria by Petropoulou and colleagues [3]. The screening process was performed in reverse chronological order, starting from the most recent ones (e.g. 31 December 2016) and working backwards until we reached 60 eligible systematic reviews per year. The screening strategy was pragmatic, as we anticipated that systematic reviews published later after the PRISMA-NMA release would be more likely to have incorporated the necessary extensions of good reporting, allegedly improving their reporting quality. Additional file 1: Table S2 illustrates the pragmatic screening process used for systematic reviews with at least three interventions published from 2016 to 2021. The list with all 721 eligible systematic reviews comprising the present study’s dataset is publicly available on figshare [27].

### Extraction process

Initially, pilot testing of the extraction form was conducted to finalise the extracted reporting items and ensure consistency in the extraction process among the involved parties. The protocol for the extraction form is publicly available on figshare [28]. The pilot testing was performed on randomly selected systematic reviews. All authors of the study (LMS, CK, JJYN, AMGS, DCRP, SES, and KP) performed the complete extraction using all 721 eligible systematic reviews. The extraction was undertaken blinded and independently in pairs of reviewers. Disagreements were resolved through discussion until a consensus was reached.

### The extracted reporting items

To determine the complete set of reporting items, we adapted Donegan and colleagues’ quality criteria for transitivity evaluation [12]. The authors originally developed the quality criteria following the recommendations from relevant publications [12]; we refined most criteria and introduced new items for extraction. In line with Donegan et al. [12], we scrutinised the systematic reviews to identify the following information: the definition of the transitivity assumption; any additional analyses employed to assess transitivity or explain the statistical heterogeneity (including sensitivity analysis, subgroup analysis, or meta-regression); and the table reporting trial and participant characteristics. We recorded the verbatim definition of the transitivity assumption, where applicable. In addition to the work by Donegan et al. [12], we sought information on the planning of the transitivity evaluation in the protocol, whether the authors inferred the plausibility of transitivity, and whether they discussed the implications for the conclusions in the systematic review. The complete set of reporting items [28] also serves as a checklist to aid researchers in ensuring a thorough and transparent reporting and evaluation of the transitivity assumption as it expands on items 14, 16, 25, and 26 of the PRISMA-NMA statement [11] to emphasise the reporting of transitivity assumption.

#### Awareness and evaluation of the transitivity assumption

In addition to defining the transitivity assumption, we sought information on whether the authors (1) explicitly stated in the methods section of the systematic review to have evaluated transitivity and (2) reported the evaluation results. Among the methods employed for the transitivity evaluation, we investigated whether the authors considered the five interchangeable interpretations (Table 1) and whether they conducted the transitivity evaluation as planned in the methods section. We categorised the methods into *direct* and *indirect* evaluation of transitivity. The direct evaluation included the statistical or narrative assessment of the comparability of the observed comparisons based on the characteristics defining the PICO (population, interventions, comparators, and outcomes) framework (item (e) in Table 1) and the remaining four interchangeable interpretations of the transitivity assumption (items (a)–(d) in Table 1). The indirect evaluation included statistical methods, such as sensitivity analysis, subgroup analysis, meta-regression, and consistency evaluation (the statistical agreement between direct and indirect evidence), which implies the statistical manifestation of transitivity [5, 29]. These reporting items demystified the awareness of the transitivity assumption and the available evaluation methods. Note that when the consistency evaluation suggests possible inconsistency, sensitivity analysis, subgroup analysis, and meta-regression may be used to improve the plausibility of transitivity and mitigate confounding bias. These statistical tools are also used to assess the sensitivity of the results to reasonable assumption changes and investigate statistical heterogeneity [29].

#### Acknowledging the implications of transitivity evaluation

We extracted information on whether the authors (1) inferred the (im)plausibility of the transitivity assumption and (2) acknowledged the implications for the interpretation and discussion of the results. We recorded the method(s) used by the authors to infer or imply the (im)plausibility of the assumption, where this information was found in the article (i.e. abstract, results, discussion, conclusion), and which NMA parameters were considered, including relative treatment effects, intervention ranking, heterogeneity parameter, and inconsistency parameter. For the systematic reviews that questioned the plausibility of the transitivity assumption, we noted whether the authors refrained from performing NMA and recorded the verbatim justification of their decision. These reporting items provided insights into the authors’ awareness of the implications of the transitivity evaluation on the credibility of the NMA results. Discussing the NMA results in the context of the transitivity evaluation increases the credibility of the conclusions drawn in the systematic review.

#### Reporting the table of characteristics

Lastly, we extracted information on the content and structure of the table of characteristics. For the systematic reviews that reported a table of characteristics, we recorded the location of the table (i.e. main body of the article, at the supplementary material, or both); the number of quantitative, qualitative, and mixed characteristics (combination of quantitative and qualitative characteristics) presented in the table; the number of participant features (e.g. demographic and clinical characteristics), intervention features (e.g. description, doses and co-interventions), outcome features (e.g. description and evaluation timepoints), and design features (e.g. country, publication year, study design, funding, conflicts of interest, duration, sample size, participant losses, risk of bias results); how the table presented the characteristics (i.e. at trial-level, at comparison-level or intervention-level with characteristics summarised across the corresponding trials, or using descriptive statistics for each characteristic); and whether there was at least one trial (or comparison) that did not report at least one of the characteristics in the table. These reporting items elucidated the quality of evaluating the transitivity assumption using the table of characteristics. Depending on the table structure and quantity of characteristics presented, the table of characteristics may facilitate or hinder the evaluation of clinical and methodological heterogeneity necessary to determine the similarity of comparisons concerning important effect modifiers (item (e) in Table 1).

### Statistical analysis and results presentation

#### Tabulation and binomial logistic regression

We summarised the extracted information in both textual and tabular formats. The textual format included quoting the definition of transitivity and the five interchangeable interpretations (Table 1) as reported in the protocol and the main body of the systematic reviews. We created tables and presented each reporting item before and after the PRISMA-NMA statement. To describe the reporting items, we used absolute and relative frequencies. We applied binomial logistic regression for each reporting item to compare the reporting quality of systematic reviews published *after versus before* the PRISMA-NMA statement. The regression results were reported as odds ratios (OR) and 95% confidence intervals (CI). We interpreted the evidence as conclusive when the corresponding 95% CI excluded an OR of 1; otherwise, the evidence was inconclusive. Specifically, an OR greater than 1 indicated improvement in reporting the corresponding item, while an OR less or equal to 1 suggested minimal reporting.

#### Ad hoc analysis on reporting completeness: 2016 versus 2021

As an ad hoc analysis, we compared the reporting completeness of two extreme cases: systematic reviews published in 2016 and those in 2021. By considering a 5-year distance after the release of the PRISMA-NMA (June 2015), we aimed to investigate whether allowing for more time after the PRISMA-NMA release would have led to improved reporting completeness of transitivity evaluation. We also checked whether these systematic reviews mentioned the PRISMA-NMA statement in their report and illustrated the results from each timeframe using a bar plot.

#### Figures and statistical software

To illustrate the distribution of the number of quantitative, qualitative, and mixed characteristics and the number of each PICO feature before and after the PRISMA-NMA statement, we constructed box plots with jitter points. Bar plots were used to present various categorical reporting items, such as the frequency of each direct and indirect evaluation method of transitivity at the protocol and systematic review levels, the most and least popular table structure, and the most and least frequent location of the table of characteristics in the systematic review. Bubble plots were created to depict the frequency of each health field before and after the statement. Additionally, bubble plots were used to present the frequency of each conclusion regarding transitivity (i.e. plausible, questionable, or difficult to judge due to limited data) before and after the PRISMA-NMA release. Finally, lollipop plots were utilised to summarise several reporting items at protocol and systematic review levels regarding their reporting frequency before and after the statement. We also used this plot to illustrate the results from the ad hoc analysis. For the analyses and figure creation, we used the statistical software R (version 4.3.0 [30]) and specifically the R-package *ggplot2* for the figures [31].

## Results

### Distribution of health fields

The systematic reviews of our collection spanned across 19 different health fields (Additional file 2: Figure S1). Cardiovascular diseases ($n=75$ out of 361;$20.8\%$) were the most prevalent among the systematic reviews published before the PRISMA-NMA statement, followed by oncology ($n=32$ out of 361;$8.9\%$) and nutrition ($n=27$ out of 361;$7.5\%$). Systematic reviews published after PRISMA-NMA were populated mostly by oncology research ($n=76$ out of 360;$21.1\%$), followed by cardiovascular diseases ($n=55$ out of 360;$15.3\%$) and gastroenterology ($n=28$ out of 360;$7.8\%$).

### Awareness and evaluation of the transitivity assumption

#### Protocol level: reporting and evaluating transitivity

Only twenty-nine per cent of the systematic reviews ($n=210$ out of 721) had an available protocol (Table 2). The percentage of systematic reviews with an available protocol increased to 42.5% ($n=153$ out of 360) after the PRISMA-NMA release compared to 15.8% ($n=57$ out of 361) before PRISMA-NMA. Systematic reviews published after PRISMA-NMA were approximately four times more likely to have made their protocol available than those published before PRISMA-NMA (OR: 3.94, 95% CI: 2.79–5.64) (Table 2).

**Table 2:** Awareness and evaluation of the transitivity in the protocol and publication of the review

Characteristic	Levels	Total (*n*=721)		Before PRISMA-NMA (*n*=361)			After PRISMA-NMA (*n*=360)		Odds ratio (95% CI)
		n	%	n	%		n	%	
*Reporting and evaluating transitivity in the protocol*									
[1] The study protocol is available^a^	Yes	210	29.1	57	15.8		153	42.5	**3.94 (2.79, 5.64)**
	No	511	70.9	304	84.2		207	57.5	
[2] If the protocol is ‘Available’ (**210** SRs), the authors defined the transitivity assumption	Yes	31	14.8	9	15.8		22	14.4	0.90 (0.40, 2.17)
	No	179	85.2	48	84.2		131	85.6	
[3] If the protocol is ‘Available’ (**210** SRs), the authors mentioned that they planned to evaluate the transitivity assumption in the review	Yes	85	40.5	13	22.8		72	47.1	**3.01 (1.54, 6.23)**
	No	125	59.5	44	77.2		81	52.9	
*Reporting and evaluating transitivity in the systematic review*									
[4] The authors defined transitivity	Yes	218	30.2	130	36.0		88	24.4	**0.57 (0.42, 0.79)**
	No	503	69.8	231	64.0		272	75.6	
[5] The authors explicitly stated in the methods that they planned to evaluate transitivity and reported the evaluation results	Yes	442	61.3	190	52.6		252	70.0	**2.10 (1.55, 2.86)**
	No	279	38.7	171	47.4		108	30.0	
[6] The authors did *not* state in the methods any plans for transitivity evaluation, but evaluation results were found in the manuscript	Yes	30	4.2	19	5.3		11	3.1	0.57 (0.26, 1.19)
	No	691	95.8	342	94.7		349	96.9	
*Acknowledging the implications of the transitivity evaluation*									
[7] The authors conclude or imply the (im)plausibility of transitivity, or the difficulty to judge	Yes	265	36.8	128	35.5	137		38.1	1.12 (0.83, 1.51)
	No	456	63.2	233	64.5	223		61.9	
[8] Among the reviews with a conclusion about transitivity (**265** SRs), some authors explicitly refrained from NMA^b^	Yes	4	1.5	1	0.8	3		2.2	2.84 (0.36, 57.89)
	No	261	98.5	127	99.2	134		97.8	
[9] Among the reviews with a conclusion about transitivity (**265** SRs), implications were discussed or implied concerning at least one NMA parameter^c^	Yes	199	75.1	105	82.0	94		68.6	**0.48 (0.27, 0.85)**
	No	66	24.9	23	18.0	43		31.4	
*Reporting the table of characteristics*									
[10] A table of characteristics is provided^d^	Yes	680	94.6	336	93.6	344		95.6	1.47 (0.77, 2.88)
	No	39	5.4	23	6.4	16		4.4	
[11] If a table of characteristics is provided (**680** SRs), the structure of the table facilitates transitivity evaluation^e^	Yes	669	98.4	331	98.5	338		98.3	0.85 (0.24, 2.85)
	No	11	1.6	5	1.5	6		1.7	
[12] Among the reviews with a proper table structure (**669** SRs), there is at least one missing characteristic across the trials or comparisons	Yes	564	84.3	272	82.2	292		86.4	1.38 (0.91, 2.10)
	No	105	15.7	59	17.8	46		13.6	

*CI* confidence interval, *NMA* network meta-analysis, *PRISMA-NMA* PRISMA extension statement for NMA, *SR* systematic review

^a^A protocol is considered available when the systematic review reports a PROSPERO number, provides the protocol as supplementary material, or has published the protocol in the same or a different Journal; otherwise, a protocol is considered not available

^b^These authors judged transitivity to be questionable or difficult to judge due to limited data (e.g. few trials, low events, poor trial reporting, missing characteristics); hence, they decided to refrain from conducting network meta-analysis

^c^The network meta-analysis parameters include the summary treatment effects, intervention hierarchy measures, statistical heterogeneity, and inconsistency evidence (i.e. the difference between direct and indirect estimates and comparing pairwise meta-analysis with network meta-analysis treatment effects for the same comparisons)

^d^There was no access to the supplementary material of two eligible articles published before the PRISMA-NMA; hence, we could not extract any necessary information related to the table of characteristics. Only for the items related to ‘Reporting the table of characteristics’, we have restricted the articles published before PRISMA-NMA to those with access to their supplementary material, namely, 359 articles

^e^Tables of characteristics that facilitate transitivity evaluation include those with a trial-level structure (i.e. trials-by-characteristic table), trials grouped by comparison with characteristics at trial-level, or comparison-level summarised characteristics. Tables of characteristics that hinder transitivity evaluation include those with intervention-level summarised characteristics or descriptive statistics for each characteristic

Of the 210 systematic reviews that provided a protocol, the majority did not define the transitivity assumption in the protocol ($n=179$;$85.2\%$), with a similar percentage observed before and after PRISMA-NMA (84.2% versus 85.6%, respectively) (Table 2). The list with the verbatim definitions of transitivity in the protocol can be found in Table S4 on figshare [32].

Furthermore, only 40.5% of the systematic reviews with an available protocol ($n=85$ out of 210) had planned to evaluate transitivity using at least one direct or indirect method (Table 2). This percentage increased to 47.1% ($n=72$ out of 153) after the PRISMA-NMA statement compared to 22.8% ($n=13$ out of 57) before PRISMA-NMA. Systematic reviews published after PRISMA-NMA were three times more likely to have provided an analysis plan for the transitivity evaluation than those published before PRISMA-NMA (OR: 3.01, 95% CI: 1.54–6.23).

**Fig. 1:**
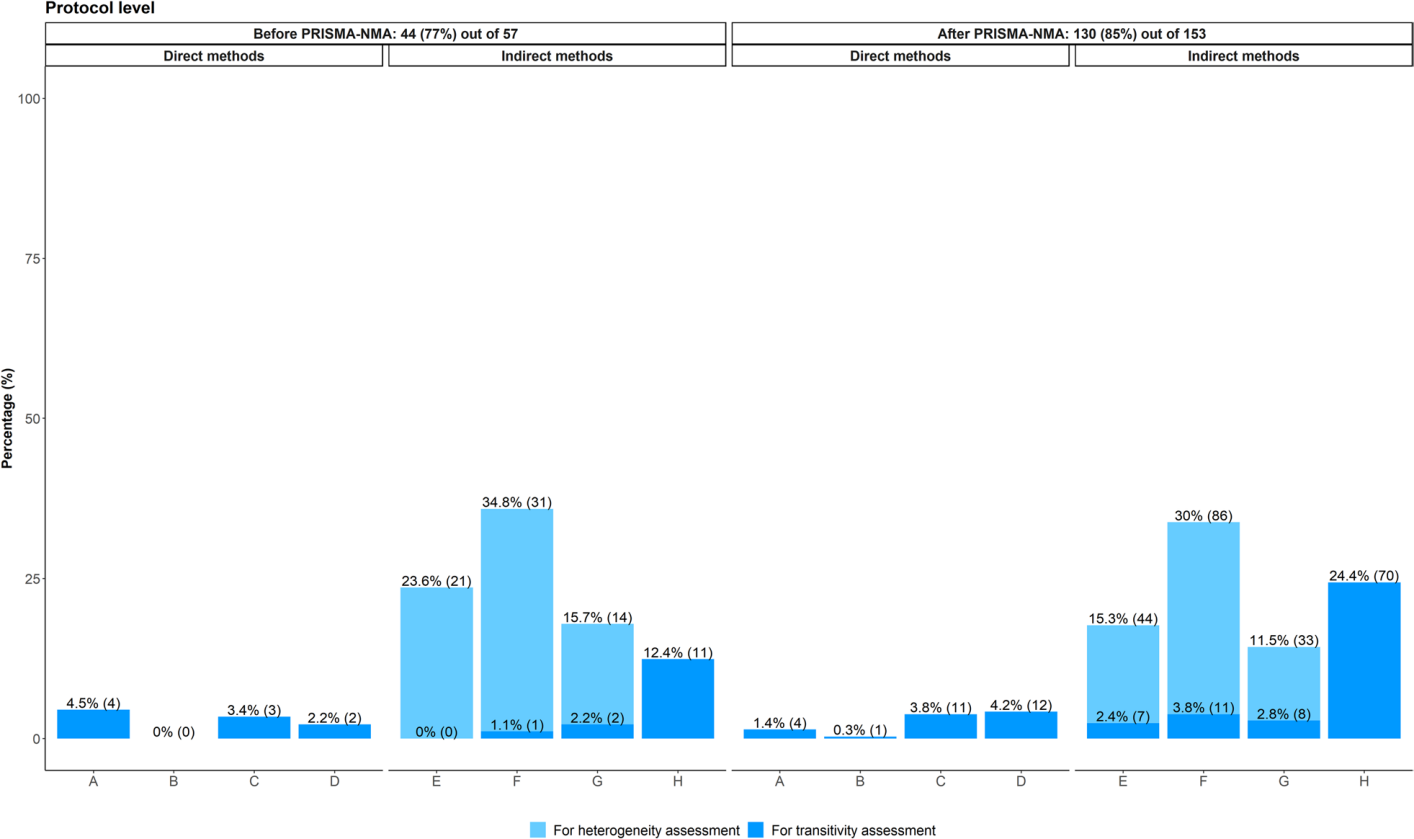
Bar plots on the methods planned in the protocol to evaluate transitivity among systematic reviews published before and after the PRISMA-NMA statement: 44 systematic reviews before and 130 after PRISMA-NMA planned *at least one* method for transitivity or statistical heterogeneity assessment. A systematic review may have planned more than one method. Dark blue refers to direct and indirect methods used exclusively for transitivity assessment. Light blue refers to indirect methods used exclusively to investigate sources of statistical heterogeneity. **A** Justifying treatment similarity in different trials. **B** Justifying treatments as missing at random. **C** Justifying participants as jointly randomisable. **D** Comparison comparability regarding important effect modifiers. **E** Sensitivity analysis. **F** Subgroup analysis. **G** Meta-regression. **H** Consistency evaluation

The verbatim justifications of the planned direct methods for transitivity evaluation can be found in Tables S5 and S6 on figshare [32]. Direct methods were the least preferred when planning the transitivity evaluation, regardless of the PRISMA-NMA statement (Fig. 1): 10.1% and 9.7% of systematic reviews before and after PRISMA-NMA, respectively, considered direct methods, compared to 15.7% and 33.4% of the systematic reviews, respectively, that opted for indirect methods to evaluate transitivity. Among the direct methods, there was a slight increase of attention to comparison comparability regarding important effect modifiers after PRISMA-NMA (4.2% from 2.2% before PRISMA-NMA), followed by justifying whether participants could be jointly randomisable (3.8% from 3.4% before PRISMA-NMA) (Fig. 1). However, less attention was given to justifying whether interventions were similar in different comparisons after PRISMA-NMA (1.4% from 4.5% before PRISMA-NMA), and almost none of the systematic reviews in both timeframes planned to justify whether treatments were missing at random (Fig. 1).

Among the indirect methods, planning to investigate sources of statistical heterogeneity was most commonly reported compared to planning to evaluate transitivity (Fig. 1): 74.1% and 56.8% of the systematic reviews before and after PRISMA-NMA, respectively, applied sensitivity analysis, subgroup analysis, or meta-regression to assess statistical heterogeneity. In contrast, 3.3% and 9.0% of the systematic reviews before and after PRISMA-NMA, respectively, applied these indirect methods to assess sources of inconsistency and increase the plausibility of transitivity. However, planning to use these indirect methods for heterogeneity assessment dropped after the PRISMA-NMA release and followed the increased planning for consistency evaluation (24.4% from 12.4% before PRISMA-NMA). Attention to sensitivity analysis, subgroup analysis and meta-regression for transitivity evaluation increased partly after the release of PRISMA-NMA, with subgroup analysis being slightly preferred (3.8% after versus 1.1% before PRISMA-NMA) (Fig. 1).

#### Systematic review level: reporting and evaluating transitivity

One out of three systematic reviews stated the notion of transitivity in their report ($n=218$ out of 721) (Table 2): the percentage was conclusively higher in systematic reviews published before the PRISMA-NMA statement (36.0%; $n=130$ out of 361) than those published after the statement (24.4%; $n=88$ out of 360) (OR: 0.57, 95% CI: 0.42–0.79). The definition of transitivity was most often reported in the methods and discussion sections of the systematic review report (Additional file 2: Figure S2 (a)). Table S7 on figshare [32] lists the verbatim definitions of the transitivity assumption and their location in the systematic review reports.

More than half of the systematic reviews (61.3%; $n=442$ out of 721) described how they evaluated transitivity in the methods section and reported the results (Table 2). Systematic reviews published after PRISMA-NMA were twice as likely to report the transitivity evaluation and results as those published before PRISMA-NMA (OR: 2.10, 95% CI: 1.55–2.86). Only 4.2% ($n=30$ out of 721) of all systematic reviews reported results from the transitivity evaluation without describing the evaluation in the methods section: the percentage was similarly low before and after PRISMA-NMA (Table 2), indicating that systematic reviews were transparent overall in reporting the methods for transitivity evaluation.

Most systematic reviews that described the transitivity evaluation in the methods section evaluated transitivity as planned (93.2% before versus 90.9% after PRISMA-NMA) (Additional file 2: Figure S3). Some systematic reviews faced challenges evaluating the transitivity assumption due to limited data (6.8% before versus 9.1% after PRISMA-NMA).

**Fig. 2:**
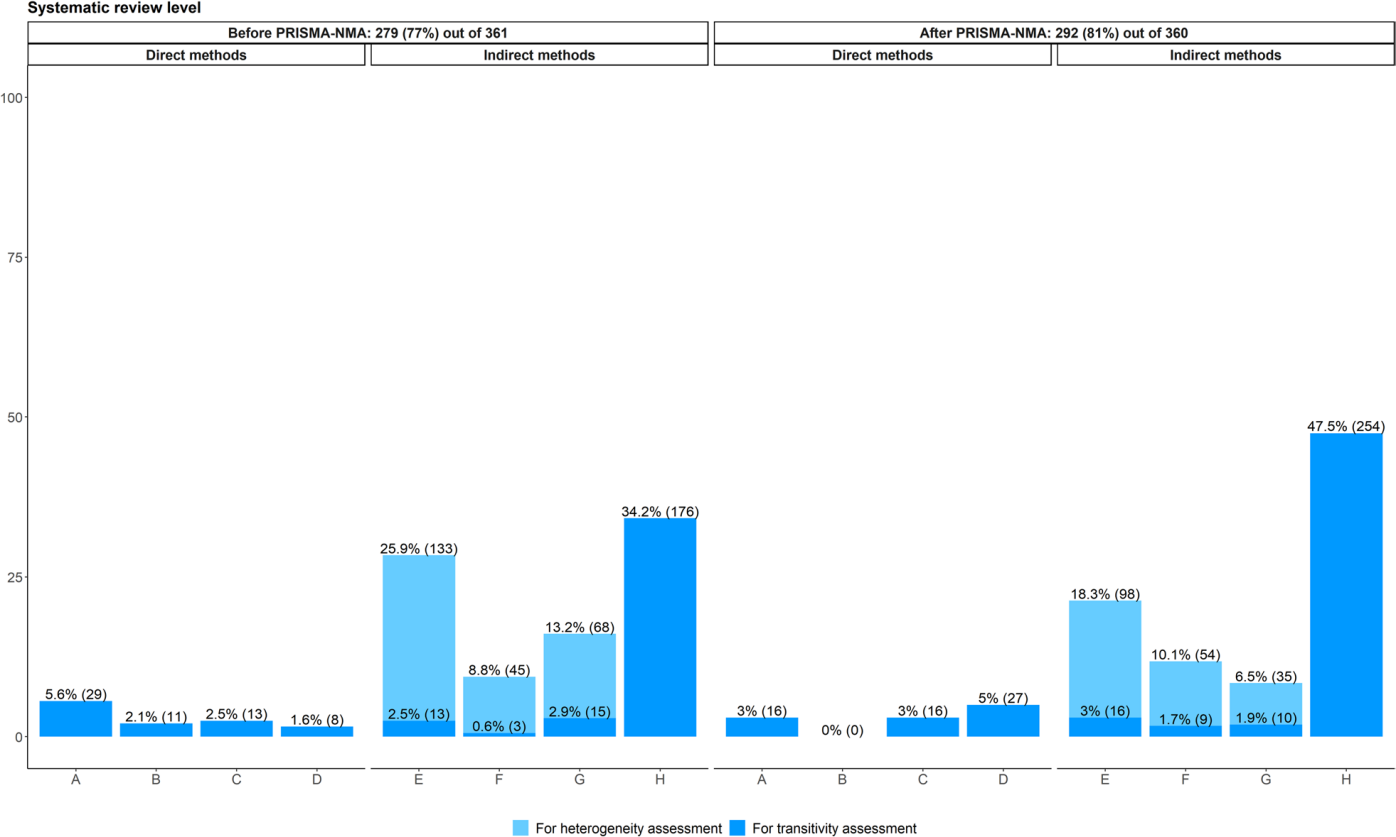
Bar plots on the methods described in the systematic review report for transitivity evaluation among systematic reviews published before and after the PRISMA-NMA statement: 279 systematic reviews before and 292 after PRISMA-NMA reported *at least one* method for transitivity or statistical heterogeneity assessment. A systematic review may have reported more than one method. Dark blue refers to direct and indirect methods used exclusively for transitivity assessment. Light blue refers to indirect methods used exclusively to investigate sources of statistical heterogeneity. **A** Justifying treatment similarity in different trials. **B** Justifying treatments as missing at random. **C** Justifying participants as jointly randomisable. **D** Comparison comparability regarding important effect modifiers. **E** Sensitivity analysis. **F** Subgroup analysis. **G** Meta-regression. **H** Consistency evaluation

Tables S8 and S9 on figshare [32] list the verbatim justifications of the reported direct methods for transitivity evaluation. In line with the protocol of the systematic reviews (Fig. 1), direct methods were the least utilised in the transitivity evaluation (11.8% before versus 11.0% after PRISMA-NMA) as opposed to the indirect methods (40.2% before versus 54.1% after PRISMA-NMA) (Fig. 2). Among the indirect methods, consistency evaluation was the most prevalent method for evaluating transitivity, particularly after PRISMA-NMA (34.2% before versus 47.5% after PRISMA-NMA) (Fig. 2). Furthermore, sensitivity analysis, subgroup analysis and meta-regression were mainly applied to investigate sources of statistical heterogeneity rather than to assess transitivity (Fig. 2): 47.9% and 34.9% of the systematic reviews before and after PRISMA-NMA, respectively, applied these indirect methods to explain the statistical heterogeneity only, as opposed to 6.0% and 6.6% of the systematic reviews, respectively, that explicitly aimed to assess sources of inconsistency.

#### Systematic review level: discussing the transitivity evaluation

Three out of eight systematic reviews discussed the results of the transitivity evaluation ($n=265$ out of 721) (Table 2), with the percentage being very similar before and after the PRISMA-NMA statement (35.5% versus 38.1%, respectively). Fifty-five per cent of the systematic reviews concluded that transitivity might be plausible ($n=145$ out of 265), followed by 29.1% ($n=77$ out of 265) that could not infer in favour of or against transitivity due to limited available data, and 16.2% ($n=43$ out of 265) that questioned the plausibility of transitivity (Additional file 2: Figure S4). A similar pattern of conclusions was observed when PRISMA-NMA was considered: 62.5% of the systematic reviews before versus 47.4% after PRISMA-NMA inferred transitivity was plausible, followed by 26.6% before versus 31.4% after PRISMA-NMA that could not conclude due to limited available data, and 10.9% before versus 21.2% after PRISMA-NMA that questioned transitivity (Additional file 2: Figure S4).

**Fig. 3:**
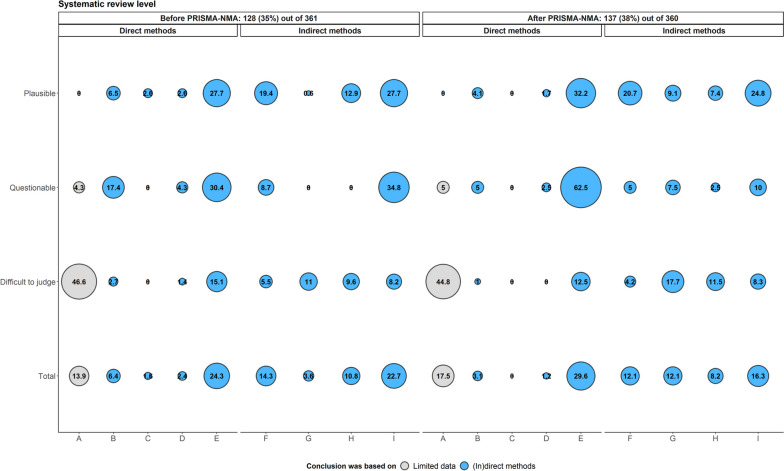
Bubble plot on the justifications considered to support the conclusions about transitivity (plausible, questionable, or difficult to judge) among systematic reviews published before and after the PRISMA-NMA statement: 128 systematic reviews before and 137 after PRISMA-NMA reported their conclusion about transitivity. A systematic review may have reported *at least one* justification (*x*-axis) to support its conclusion (*y*-axis). **A** Limited available data. **B** Justifying treatment similarity in different trials. **C** Justifying treatments as missing at random. **D** Justifying participants as jointly randomisable. **E** Comparison comparability regarding important effect modifiers. **F** Sensitivity analysis. **G** Subgroup analysis. **H** Meta-regression. **I** Consistency evaluation

Generally, systematic reviews mostly mentioned the comparability of trials or treatment comparisons when discussing the transitivity evaluation (24.3% before versus 29.6% after PRISMA-NMA), followed by the consistency evaluation (22.7% before versus 16.3% after PRISMA-NMA) and the limited available data (13.9% before versus 17.5% after PRISMA-NMA) (‘Total’ category in Fig. 3). Trial or comparison comparability and consistency evaluation were considered the most frequently reported factors in supporting conclusions regarding the plausibility or questioning of transitivity in systematic reviews before and after PRISMA-NMA (‘Plausible’ and ‘Questionable’ categories in Fig. 3). At the same time, the limited available data was the main argument for the respective conclusions (‘Difficult to judge’ category in Fig. 3).

Among the 120 systematic reviews that questioned or found it difficult to postulate transitivity (Additional file 2: Figure S4), only one systematic review before PRISMA-NMA and three after the statement explicitly stated to have refrained from conducting NMA (Table 2). Limited available data, limited trial comparability concerning clinical and methodological heterogeneity, and statistically significant inconsistency comprised the reasons these systematic reviews did not pursue NMA. The verbatims of the systematic reviews that refrained from NMA can be found in Additional file 1: Table S3 [33–36].

One hundred ninety-nine (75.1%) out of 265 systematic reviews with a conclusion about transitivity considered at least one NMA parameter (Table 2). Systematic reviews published before PRISMA-NMA were conclusively more likely to include NMA parameters in their discussion about transitivity (82.0%; $n=105$ out of 128) compared to systematic reviews after PRISMA-NMA (68.6%; $n=94$ out of 137) (OR: 0.48, 95% CI: 0.27–0.85) (Table 2). Specifically, the summary treatment effects were the most preferred NMA parameter (45.4% before versus 51.2% after PRISMA-NMA), followed by consistency evaluation (27.0% before versus 22.0% after PRISMA-NMA), statistical heterogeneity (19.5% before versus 15.4% after PRISMA-NMA), and intervention ranking (8.0% before versus 11.4% after PRISMA-NMA) (Additional file 2: Figure S5). Most systematic reviews used the discussion section to confer transitivity (76.7% before versus 90.3% after PRISMA-NMA) (Additional file 2: Figure S2 (b)).

#### Systematic review level: reporting the table of characteristics

Almost all systematic reviews provided a table of characteristics, populated with several participant, outcome, intervention, and design features (94.6%; $n=680$ out of 719) (Table 2): 93.6% ($n=336$ out of 359) before and 95.6% ($n=344$ out of 360) after PRISMA-NMA reported that table. The structure of the table of characteristics facilitated the transitivity evaluation, particularly regarding the comparability of treatment comparisons, in almost all systematic reviews (98.4%; $n=669$ out of 680) (Table 2, and Additional file 2: Figure S6 (a)): 98.5% and 98.3% of the systematic reviews published before and after PRISMA-NMA, respectively, considered such a table (Table 2). Most systematic reviews reported the characteristics at the trial level (i.e. the characteristics occupied the columns, and the trials occupied the rows of the table) (84.5% before versus 94.2% after PRISMA-NMA), followed by stratifying the trials by comparison (13.1% before versus 2.6% after PRISMA-NMA) (Additional file 2: Figure S6 (a)). Three systematic reviews before and five after PRISMA-NMA (0.9% versus 1.5%) tabulated the summary statistics of each quantitative characteristic by comparison (Additional file 2: Figure S6 (a)). Table structures that hindered transitivity evaluation included tabulating the summary statistics of each quantitative characteristic by the intervention ($n=4$ out of 336 before versus $n=2$ out of 344 after PRISMA-NMA) and presenting summary statistics for each characteristic ($n=1$ out of 336 before versus $n=4$ out of 344 after PRISMA-NMA) (Additional file 2: Figure S6 (a)).

Among the systematic reviews with a properly structured table of characteristics, approximately six out of seven reported having at least one missing characteristic across the trials or comparisons in the table ($84.3\%$; $n=564$ out of 669), with the percentage being similar before and after the PRISMA-NMA release (82.2% versus 86.4%, respectively) (Table 2). Of the 680 systematic reviews with a table of characteristics, the majority reported the table in the main body of the article (67.6% before versus 63.7% after PRISMA-NMA), followed by supplementary material (28.0% before versus 33.4% after PRISMA-NMA) (Additional file 2: Figure S6 (b)).

**Fig. 4:**
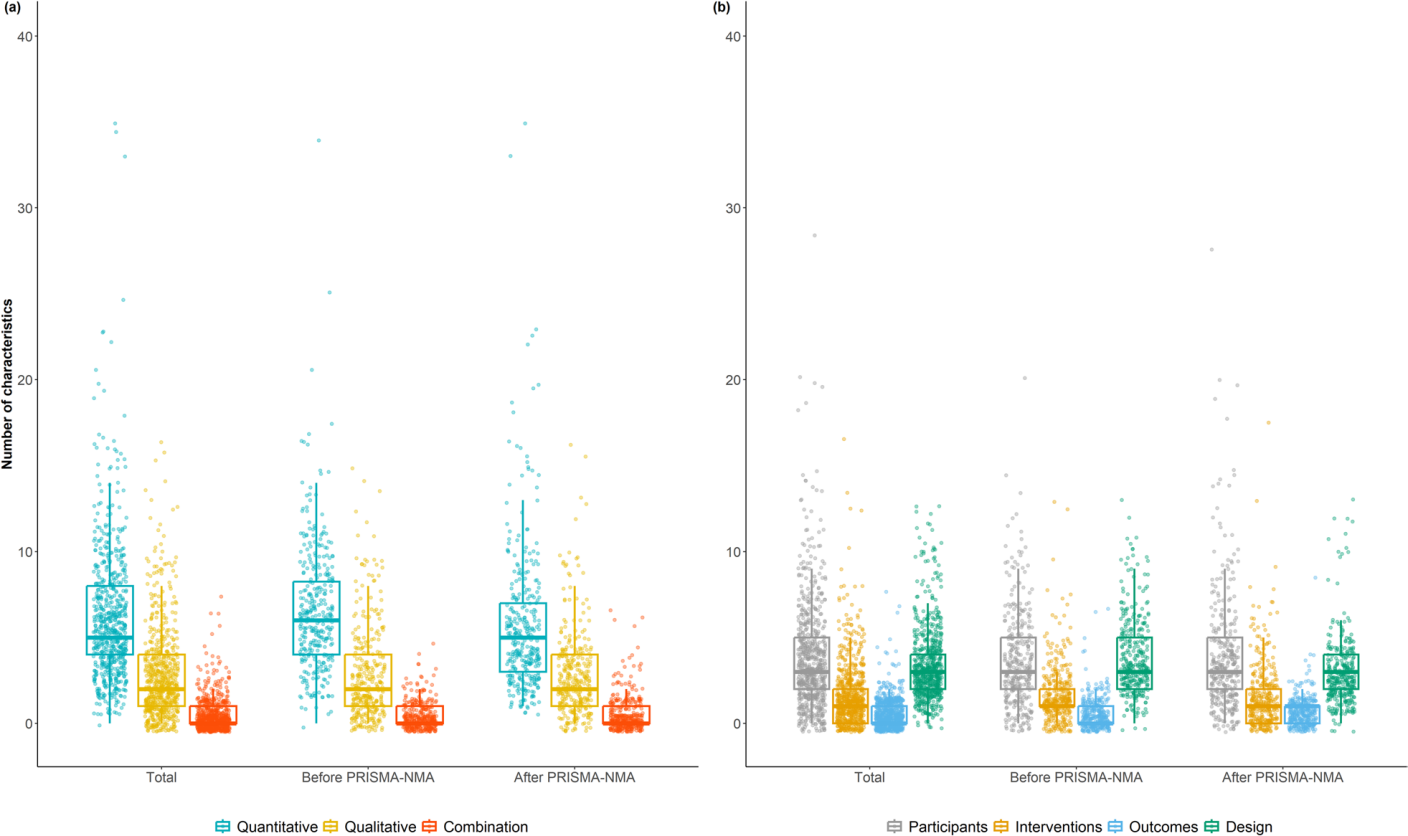
Box plots with incorporated jitter dots on the number of quantitative, qualitative, and mixed characteristics (plot (**a**)) and the number of participant, intervention, outcome, and design characteristics (plot (**b**)) reported in the table of characteristics of systematic reviews published before and after the PRISMA-NMA statement

The number of characteristics included in the table ranged from 0 to 35 (median: 2, interquartile range: 0 to 5) across the systematic reviews (Fig. 4a). Most of the characteristics were quantitative, followed by qualitative, with this pattern being consistent before and after PRISMA-NMA. When distinguishing among the PICO components that populated the table of characteristics, various participant features were the most prevalent, followed by design and intervention features, with the same trend before and after PRISMA-NMA (Fig. 4b).

### Improvements and gaps in reporting and evaluating transitivity

**Fig. 5:**
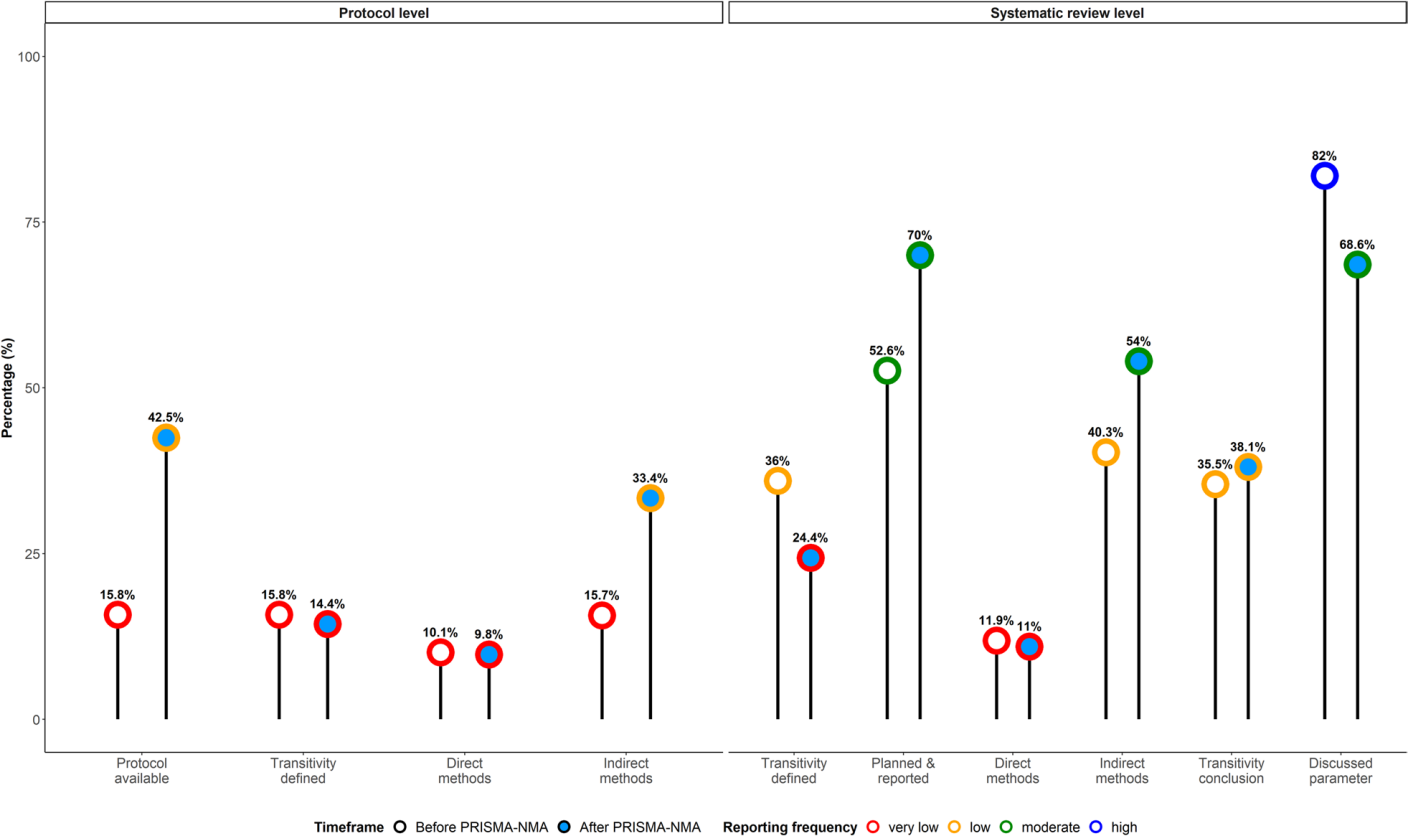
Lollipop plot summarising the reporting frequency of the reporting items to determine gaps and improvements in reporting and evaluating the transitivity assumption among systematic reviews published before and after the PRISMA-NMA statement. Percentage frequency below 25% is *very low*, equal or above 25% but below 50 is *low*, equal or above 50% but below 75% is *moderate*, and at least 75% is *high*. The percentage of direct and indirect methods for transitivity has been calculated using the subset of systematic reviews that reported *at least one* direct or indirect method. The percentage of transitivity definition at the protocol level has been calculated using the subset of systematic reviews that made a protocol available

Figure 5 summarises the results of the reporting items before and after PRISMA-NMA, distinguishing between the items that have shown improvement and those that required more attention in reporting and evaluating transitivity. The reporting items associated with the systematic review protocol were less frequently utilised overall. Although protocol availability and indirect method application have increased since the PRISMA-NMA release, their reporting frequency was low (43% and 33%, respectively). Describing the notion of transitivity and adopting direct methods to evaluate transitivity comprised major gaps in reporting and evaluating transitivity at the protocol level for having a very low reporting frequency since the PRISMA-NMA release (14% and 9%, respectively).

At the systematic review level, there was an overall increase in the frequency of the related reporting items (Fig. 5). Specifically, there has been an increased implementation of indirect methods and transparency in reporting the evaluation methods and results since the PRISMA-NMA release (54% and 70%, respectively). Discussing the results of or challenges with transitivity evaluation maintained a low frequency at 38%; acknowledging the implications on the NMA parameters displayed an improved yet, moderate reporting frequency at 69% since the PRISMA-NMA release. In line with the evidence at the protocol level, the reporting frequency for describing transitivity was very low at 24%, and that for using direct methods was even lower at 11% after PRISMA-NMA, requiring immediate attention. Discussing the implications of transitivity evaluation on the NMA parameters before PRISMA-NMA was the only item with a high reporting frequency at 82%.

#### Ad hoc analysis on reporting completeness: 2016 versus 2021

**Fig. 6:**
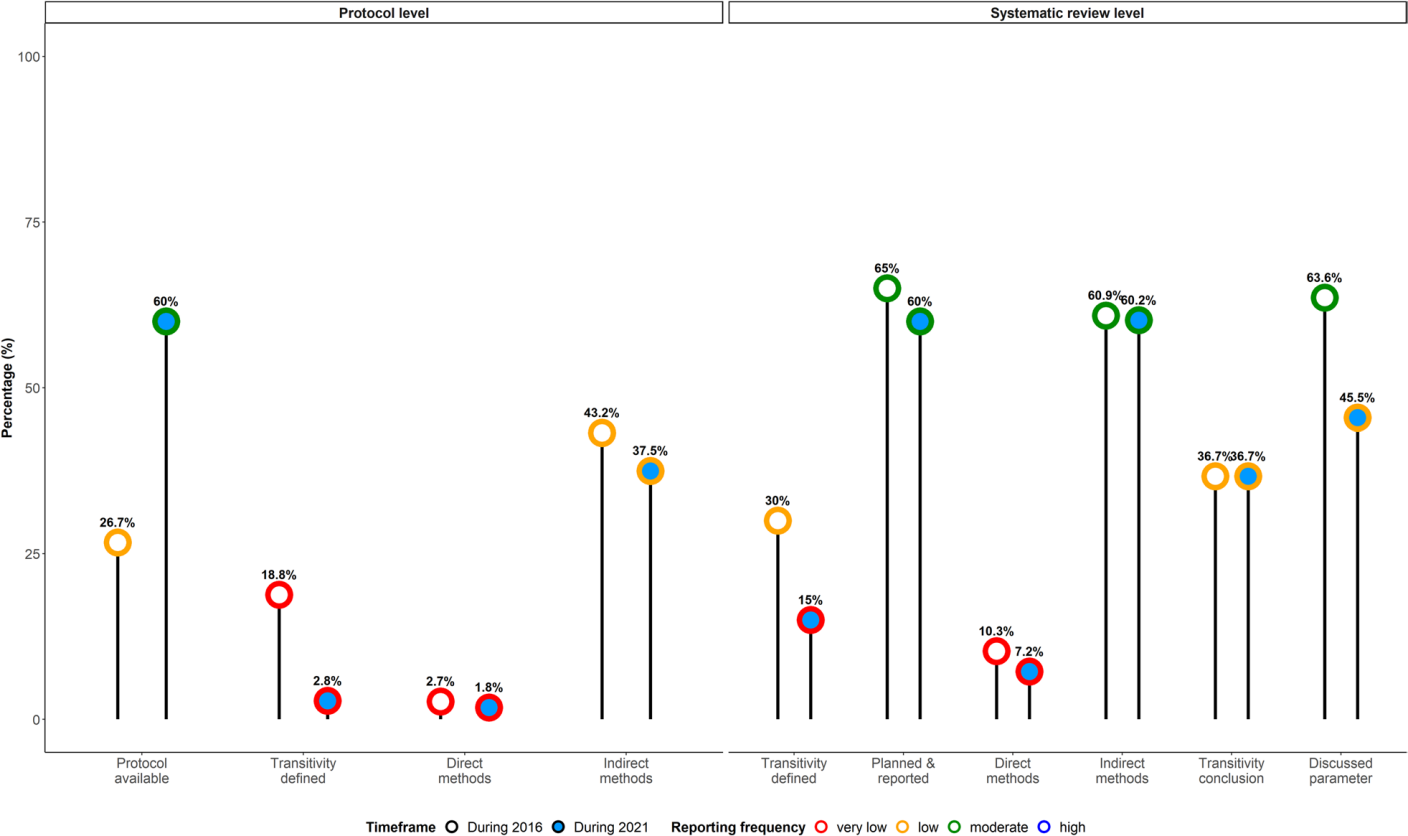
Lollipop plot summarising the reporting frequency of the reporting items to determine gaps and improvements in reporting and evaluating the transitivity assumption among systematic reviews published during 2016 and 2021. Percentage frequency below 25% is *very low*, equal or above 25% but below 50 is *low*, equal or above 50% but below 75% is *moderate*, and at least 75% is *high*. The percentage of direct and indirect methods for transitivity has been calculated using the subset of systematic reviews that reported *at least one* direct or indirect method. The percentage of transitivity definition at the protocol level has been calculated using the subset of systematic reviews that made a protocol available

Overall, the ad hoc analysis revealed similar patterns with those observed by analysing all 721 systematic reviews (Fig. 6): protocol availability was higher during 2021, reaching moderate levels (60% versus 27%). Transitivity definition and implementation of direct methods had very low reporting frequency in protocol and review reports (Fig. 6). Interestingly, planning indirect methods in protocol was slightly higher, but at low reporting frequency, during 2016 (43% versus 38%). Planning and reporting of transitivity evaluation in the review report were similarly distributed with a moderate reporting frequency in both periods (65% versus 60% during 2016 and 2021, respectively). Both periods almost coincided regarding the reporting frequency of indirect methods (61%) and had the same low reporting frequency for transitivity conclusions (37%). Parameter discussion showed the same tendency as that from analysing all systematic reviews, though, at moderate and low levels for 2016 and 2021, respectively.

PRISMA-NMA was more frequently mentioned among the systematic reviews published in 2021 than those published in 2016 (58.3% versus 21.7%; Additional file 2: Figure S7 [24, 37, 38]). In contrast, almost half of the systematic reviews published in 2016 did not mention if they were reported according to a PRISMA statement. However, the completeness of transitivity reporting among systematic reviews published in 2021 did not align with the observed popularity of PRISMA-NMA since 7 in 10 reporting items were associated with very low and low reporting frequency (Fig. 6), necessitating the scrutiny of the systematic reviews to understand whether they employed the PRISMA-NMA statement properly.

## Discussion

This study provides comprehensive empirical evidence on reporting and evaluating the transitivity assumption in systematic reviews before and after the PRISMA-NMA release. The evidence is overall underwhelming with a partial improvement in certain reporting items since PRISMA-NMA but low awareness of the evaluation methods. The transitivity assumption and its evaluation remain elusive to most users of NMA. Systematic reviews showed limited emphasis on direct methods, which are crucial for transitivity evaluation for aligning with the conceptual nature of the assumption. Infrequent descriptions of the transitivity notion at the protocol and systematic review levels may have led to the low application of direct methods.

Our study found a more frequent definition of transitivity in systematic reviews published before the PRISMA-NMA release than those after (Fig. 5 and item 4 in Table 2). This trend may be attributed to the increased emphasis on consistency assessment and investigation of possible effect modifiers since Donegan and colleagues’ publication [12], potentially overshadowing the relevance of transitivity [2, 5, 8, 29]. The lack of an established transitivity evaluation framework, unlike the extensive literature on consistency and effect modification over time [2, 5, 8, 29], likely contributed to the infrequent definition of transitivity in post-PRISMA-NMA systematic reviews. A methodological framework for transitivity evaluation could involve reformulating some of the interchangeable interpretations in Table 1 into testable hypotheses (e.g. item e)), employing unsupervised methods to explore similarity in the participant, interventions and trial characteristics and incorporating mixed methods to address the qualitative nature of the transitivity assumption properly.

Of the 721 systematic reviews analysed, only four explicitly refrained from conducting NMA after evaluating transitivity (item 8 in Table 2). This remarkably low figure raises serious concerns regarding the conclusions presented to end-users. It also underscores the authors’ limited awareness of intransitivity implications. While transitivity assessment and reporting are crucial, pursuing NMA when transitivity is uncertain or challenging due to evidence limitations could yield meaningless results with harmful decision-making implications.

There was a misconception regarding appropriately investigating the comparability of treatment comparisons with respect to the distribution of effect modifiers (Tables S6 and S9 on figshare [32]). Of the 89 systematic reviews reporting this direct method, most compared trials on effect modifiers and employed statistical methods to assess trial similarity within comparisons, or it was unclear whether the evaluation pertained to trials, interventions, or comparisons of interventions ($60.7\%$; $n=54$ out of 89) (Table S9 on figshare [32]). However, demonstrating similarity across trials, interventions, or within comparisons is insufficient to conclude transitivity. Transitivity depends on the distribution of the effects modifiers *across* observed comparisons [13]. Trials may be homogeneous within comparisons, but the comparisons may differ on average regarding the PICO features, indicating possible intransitivity. Alternatively, trials may differ within comparisons, with comparisons being similar on average, suggesting possible transitivity in the network.

Our findings aligned with those of Donegan et al. [12] on the frequency of stating the transitivity assumption (25.6% versus 30.2% in our study) and reporting a table of characteristics (88.4% versus 94.6% in our study). Donegan et al. [12] reported that 44.2% of the systematic reviews used sensitivity analysis, subgroup analysis, or meta-regression for transitivity assessment, and 25.6% compared participant or trial characteristics across trials. In our study, these percentages were lower: 6.0% before and 6.5% after PRISMA-NMA for the former and 1.6% before and 5.0% after the statement for the latter (Fig. 2). This difference may be attributed to associating the transitivity assessment mostly with consistency evaluation, as most networks in our study included closed loops of interventions. The statistical methodology for consistency evaluation intensified and established after 2010, owing to available statistical software. Notably, none of the systematic reviews retrieved by Donegan et al. [12] described the transitivity evaluation in the methods section. In our study, most systematic reviews mentioned at least one evaluation method in the methods section and reported the results. The small sample of systematic reviews in Donegan et al. [12] may explain this discrepancy. If they had considered systematic reviews with more than three interventions, they might have obtained a larger sample, likely detecting reviews describing the transitivity evaluation in the methods section.

Petropoulou et al. [3] assessed whether and how researchers evaluated the transitivity assumption and what conclusions they drew. For the overlapping period of 2011 to 2015, the authors found that 24.6% of the systematic reviews reported that transitivity might hold, aligning with our findings (22.2%; 80 out of 361; Additional file 2: Figure S4) [3]. Our results agree with the authors’ that most systematic reviews did not discuss transitivity: 74.3% (263 out of 354) in Petropoulou et al. [3] versus 64.5% (233 out of 361; item 7 in Table 2) in our study. Veroniki et al. [4] evaluated the reporting quality of 1144 systematic reviews with NMA before and after the PRISMA-NMA statement. While our study does not directly compare to theirs, as they focused on overall reporting completeness based on the PRISMA items, we also observed a *slight* improvement in reporting completeness post PRISMA-NMA, particularly for the transitivity assumption component.

The table of characteristics plays an important role in facilitating or hindering the evaluation of the transitivity assumption with respect to the comparability of the characteristics (effect modifiers) across comparisons. A table that organises trials by treatment comparison and predominantly reports quantitative and qualitative characteristics supports both conceptual and statistical aspects of the transitivity evaluation. This structure aligns with the PRISMA-NMA statement’ recommendation (item 18: ‘Study Characteristics’ in [11]). A table summarising the characteristics at the comparison level also facilitates the evaluation of the transitivity assumption. On the contrary, a table emphasising textual characteristics, including unreported characteristics in most trials, or summarising the characteristics at the intervention level complicates the transitivity evaluation.

Direct methods for transitivity evaluation, outlined in Table 1, do not necessarily depend on data availability, except when statistically assessing the comparability of comparisons in terms of important effect modifiers. In contrast, indirect methods have limitations when insufficient trials inform the comparisons or when the network does not contain closed loops of interventions. In such cases, conceptual evaluation of transitivity using interchangeable interpretations (Table 1) is essential. Despite the importance of the direct methods, our findings revealed that they received less attention than indirect methods, with the latter holding the spotlight in the published literature and statistical software [2, 5, 8, 29]. PRISMA-NMA also promotes direct methods for transitivity evaluation through examples and accompanying explanations. To promote *direct* evaluation methods, collaborative efforts among clinical experts are needed to develop guidelines and methodological research for selecting interventions and proper effect modifiers. Emphasis should be placed on the importance of a transitive network of interventions supported by examples from clinical practice and relevant literature.

The present study has several strengths; comprising 721 systematic reviews, it is the most comprehensive empirical investigation of the reporting and evaluation quality of the transitivity assumption. We assessed the researchers’ awareness of both conceptual and statistical methods examining the impact of the PRISMA-NMA statement on the completeness of reporting and evaluating transitivity. Following relevant methodological literature, we developed a set of reporting items applicable at the protocol-writing phase and throughout the conduct and reporting of a new systematic review. Our framework aids in gauging NMA feasibility and complements existing guidelines [17, 24, 38–40], as well as the PRISMA-NMA statement [11] for a more in-depth and transparent reporting and evaluation of transitivity.

A limitation of our study is our pragmatic approach to collecting systematic reviews published after the PRISMA-NMA statement, driven by project timelines amid the exponential growth of such reviews [4]. This approach, though, may have missed some relevant reviews. However, we do not anticipate that including all eligible systematic reviews published after the statement would have materially changed the trend of our results. Furthermore, we attempted to determine the conclusions of most researchers on transitivity, relying on a subjective evaluation to some extent, as our judgements hinged on the clarity of the systematic review reports. Additionally, insufficient information in a few systematic reviews hampered our judgement as to whether the researchers conducted sensitivity or subgroup analysis. Finally, we did not check whether our collection of systematic reviews published after the PRISMA-NMA release mentioned to have been PRISMA-NMA compliant since this would also require scrutinising all reports to judge the reporting completeness of PRISMA-NMA, which was out of our scope.

## Conclusions

Despite conclusive evidence in certain Table 2 items, there is limited awareness regarding appropriately reporting and evaluating the transitivity, which raises concerns about the quality of the conclusions drawn from systematic reviews. The lack of a methodological framework for transitivity evaluation and clear guidance on the consequences of intransitivity and how systematic reviewers should respond may partly contribute to this low awareness. It is essential for systematic reviewers to always register their protocol to international repositories, such as PROSPERO. In the protocol, they should outline direct and indirect methods, emphasising the former if limited data prohibit the application of indirect methods. Planning the transitivity evaluation, reporting its results, and documenting any challenges during the process should be integral to the systematic review report. Efforts should be put into justifying the inclusion or exclusion of some interventions and doses and whether the investigated network contains a jointly randomisable population, as these considerations would determine the feasibility of NMA [11].

Depending on data availability (i.e. enough trials per comparison and fully reported effect modifiers) and provided that the network connectivity is not compromised, explicit statements should be made regarding the implementation of network meta-regression, subgroup analyses, and sensitivity analyses to investigate important effect modifiers as possible sources of statistical heterogeneity *and* inconsistency. Statements on the plausibility of transitivity and its implications on the quality of NMA results should also be made explicit and accompanied by the results of the direct and indirect methods employed and the associated NMA parameters. If the feasibility of the NMA cannot be ensured, systematic reviewers should justify their decision to refrain from NMA or synthesise only a part of the network, following a transparent transitivity evaluation.

Finally, careful consideration should be given to the table of characteristics; presenting the characteristics at the trial level, grouped by comparison, aids in assessing the relevance of trials to the research question and investigating clinical and methodological heterogeneity. Summarising the characteristics at the comparison level in tabular or graphical format, such as box and bar plots, aids in the evaluation of transitivity.

### Supplementary Information

**Additional file 1: Table S1.** Results of the nmadb database [26] and a previous empirical study [25] of systematic reviews published between 01/2011 and 04/2015. **Table S2.** Results of pragmatic searches of systematic reviews published between 2016 and 2021. **Table S3.** List of verbatim on refraining from conducting network meta-analysis.

**Additional file 2: Figure S1.** Bubble plot on the distribution of the health fields among systematic reviews published before and after the PRISMA-NMA statement. **Figure S2.** Bar plots on location in the systematic review report where the transitivity notion was found (plot (a)) and where conclusions about transitivity were found (plot (b)) among systematic reviews published before and after PRISMA-NMA. **Figure S3.** Bar plots on whether transitivity was evaluated as planned among systematic reviews published before and after the PRISMA-NMA that planned transitivity evaluation in the methods section. **Figure S4.** Bar plots on the conclusions regarding transitivity among systematic reviews published before and after the PRISMA-NMA statement that discussed transitivity. **Figure S5.** Bar plots on the parameters considered when discussing the implications of transitivity evaluation on the network meta-analysis results among systematic reviews published before and after the PRISMA-NMA statement. **Figure S6.** Bar plots on the structure of the table of characteristics reported in systematic reviews (plot (a)) and the location in the systematic review report where the table of characteristics was found (plot (b)) among systematic reviews reported in systematic reviews published before and after the PRISMA-NMA statement. **Figure S7.** Bar plots on whether (and which) PRISMA statement was mentioned in the report among the 60 systematic reviews published in 2016 and those in 2021.

## Data Availability

The data that support the findings of this study are available online at https://github.com/LoukiaSpin/Empirical-study-transitivity-assumption-evaluation.git. The protocol for the extraction form is publicly available on figshare (10.6084/m9.figshare.23618037.v1). The list of the included systematic reviews is publicly available on figshare (10.6084/m9.figshare.23618040.v1). The list of verbatims is publicly available on figshare (10.6084/m9.figshare.23618043.v2).

## Abbreviations

CI: Confidence interval
NMA: Network meta-analysis
OR: Odds ratio
PICO: Population, interventions, comparators, and outcomes
PRISMA-NMA: Preferred Reporting Items for Systematic Reviews and Meta-analyses extension statement for network meta-analysis
