# Supplementary material for: Low awareness of the transitivity assumption in complex networks of interventions: a systematic survey from 721 network meta-analyses
Source: BMC Med. 2024 Mar 13;22:112. doi: 10.1186/s12916-024-03322-1 (PMC10935945; doi:10.1186/s12916-024-03322-1)
Supplement: Supplementary file 1 — Additional file 1: Table S1. Results of the nmadb database [26] and a previous empirical study [25] of systematic reviews published between 01/2011 and 04/2015. Table S2. Results of pragmatic searches of systematic reviews published between 2016 and 2021. Table S3. List of verbatim on refraining from conducting network meta-analysis. [file 12916_2024_3322_MOESM1_ESM.docx]

## Additional file 1

**Supplementary material for the manuscript entitled 'Low awareness of the transitivity assumption in complex networks of interventions: a systematic survey from 721 network meta-analyses'**

Loukia M. Spineli^1^, Chrysostomos Kalyvas^2^, Juan J. Yepes-Nuñez^3,4^, Andrés Mauricio García Sierra^3,5^, Diana C. Rivera-Pinzón^3^, Svenja E. Seide^6^, Katerina Papadimitropoulou^7^

^1^Midwifery Research and Education Unit, Hannover Medical School, Hannover, Germany

^2^Biostatistics and Research Decision Sciences, MSD Europe Inc., Brussels, Belgium

^3^School of Medicine, Universidad de los Andes, Bogotá, Colombia

^4^Pulmonology Service, Internal Medicine Section, Fundación Santa Fe de Bogotá University Hospital, Bogotá, Colombia

^5^School of Global Health Management and Informatics, University of Central Florida, Orlando, USA

^6^Institute of Medical Biometry, University Hospital Heidelberg, Heidelberg, Germany

^7^Health Economics and Market Access, Amaris Consulting, Lyon, France

**Table S1. Results of the *nmadb* database [26] and a previous empirical study [25] of systematic reviews published between 01/2011 and 04/2015**

| **Selection process** | **2011** | **2012** | **2013** | **2014** | **2015** |
| --- | --- | --- | --- | --- | --- |
| *nmadb database* | | | | | |
| **Found in the database** | **53** | **59** | **95** | **103** | **43** |
| **Total excluded for the following reasons:** | **5** | **4** | **7** | **4** | **2** |
| *Misclassified in the publication year* | 0 | 0 | 3^1^ | 3^2^ | 2^3^ |
| *Commentary* | 1 | 0 | 0 | 0 | 0 |
| *Articles in a non-English language (Chinese, Hungarian)* | 1 | 0 | 1 | 0 | 0 |
| *NMA includes non-randomised or observational studies* | 1 | 2 | 0 | 0 | 0 |
| *Cost-effectiveness studies based on published NMA results* | 0 | 0 | 1 | 0 | 0 |
| *Methodological studies* | 1 | 1 | 1 | 1 | 0 |
| *Not an NMA* | 1 | 0 | 0 | 0 | 0 |
| *Cannot locate in PubMed or Google* | 0 | 1 | 0 | 0 | 0 |
| *No new study for update since 2011* | 0 | 0 | 1 | 0 | 0 |
| **Total eligible from the *nmadb* database** | **48** | **55** | **88** | ***103^4^*** | ***44^5^*** |
| *Previous empirical study* | | | | | |
| **Found in the previous study (not included in *rnmadb*)** | **6** | **3** | **1** | **12** | **1** |
| **Total eligible** | **54** | **58** | **89** | **115** | **45** |

NMA, network meta-analysis

^1^Three articles were published in 2014.

^2^Three articles were published in 2015.

^3^Two articles were published in 2014 and 2016, respectively.

^4^This number results from including four misclassified studies (three from 2013 and one from 2015): [103 (found) – 4 (excluded)] + 4 (misclassified from 2013 and 2015)].

^5^This number results from including three misclassified studies from 2014: [43 (found) – 2 (excluded)] + 3 (misclassified from 2014)].

**Table S2. Results of pragmatic searches** **of systematic reviews published between 2016 and 2021**

| **Selection process** | **2016** | **2017** | **2018** | **2019** | **2020** | **2021** |
| --- | --- | --- | --- | --- | --- | --- |
| **Total retrieved** | **93** | **107** | **181** | **118** | **171** | **114** |
| **Total excluded for the following reasons:** | **33** | **47** | **121** | **58** | **111** | **54** |
| *Commentary, editorial, erratum, duplication, brief data* | 7 | 5 | 11 | 3 | 8 | 8 |
| *Articles in a non-English language (Chinese, Spanish, Italian)* | 0 | 0 | 4 | 1 | 4 | 3 |
| *NMA includes non-randomised or observational studies* | 5 | 16 | 19 | 17 | 20 | 7 |
| *Includes diagnostic studies* | 0 | 0 | 10 | 3 | 2 | 1 |
| *Cost-effectiveness studies based on published NMA results* | 0 | 0 | 4 | 0 | 0 | 0 |
| *Methodological studies* | 8 | 5 | 10 | 0 | 4 | 4 |
| *Gene studies, animal studies* | 0 | 2 | 13 | 1 | 2 | 0 |
| *Invalid NMA (disconnected networks)* | 0 | 1 | 0 | 1 | 0 | 0 |
| *Not an NMA* | 1 | 0 | 17 | 0 | 6 | 0 |
| *The number of trials is smaller than the number of interventions* | 2 | 5 | 2 | 2 | 6 | 1 |
| *Protocols* | 6 | 4 | 19 | 10 | 36 | 22 |
| *No access could be granted* | 4 | 9 | 12 | 20 | 23 | 8 |
| **Total eligible** | **60** | **60** | **60** | **60** | **60** | **60** |

NMA, network meta-analysis

**Table S3. List of verbatim on refraining from conducting network meta-analysis**

| **ID^1^** | **Quotation** | **Found in** |
| --- | --- | --- |
| [33] | “The heterogeneity of pre-post pain differences in the control groups of drug studies (Supporting Information Figs. S13, S15 and S17) did not allow the definition of a common comparator. Thus, basic requirements for adjusted indirect comparisons between drug categories were not met.” | Results |
| [34] | “No network analysis was carried out for the secondary endpoint of walking ability owing to statistically relevant inconsistency; the central precondition of transitivity was infringed.”  “No statements were made with regard to achievement of walking ability. We refrained from statistical evaluation because of the clear statistical inconsistency in the evidence network (26). The individual studies, the interventions used, and the patient characteristics were therefore described qualitatively instead (eTable 3).” | Results  Discussion |
| [35] | “Reporting of AEs differed between studies and led to a limited comparability. At the protocol stage, we decided to use the most frequently reported way for network meta-analysis (amount of participants with at least one event grade R 3 (or at least one SAE)). Therefore, to be able to include reported data into network meta-analyses, we could only consider AEs, when the amount of participants with at least one event grade at least 3 (or at least one SAE) were reported. We could not consider cumulated events or breakdowns in degrees of severity or further subgroups. As a result, network meta-analysis was not possible for the outcome grade 3 and 4 AEs due to severe inconsistency.” | Discussion |
| [36] | “We performed pairwise meta-analyses and indirect comparisons, where possible, but due to the limited number of included studies, we were unable to conduct the planned network meta-analysis.”  “Because of the limited number of trials, It was not possible to perform the planned network meta-analysis and rank the available uterotonic agents. [...] oxytocin. We have performed only one indirect comparison between misoprostol plus oxytocin and misoprostol alone with the common comparator being oxytocin.” | Abstract  Discussion |

^1^Reference number in the main manuscipt.
