# Supplementary material for: Low awareness of the transitivity assumption in complex networks of interventions: a systematic survey from 721 network meta-analyses
Source: BMC Med. 2024 Mar 13;22:112. doi: 10.1186/s12916-024-03322-1 (PMC10935945; doi:10.1186/s12916-024-03322-1)
Supplement: Supplementary file 2 — Additional file 2: Figure S1. Bubble plot on the distribution of the health fields among systematic reviews published before and after the PRISMA-NMA statement. Figure S2. Bar plots on location in the systematic review report where the transitivity notion was found (plot (a)) and where conclusions about transitivity were found (plot (b)) among systematic reviews published before and after PRISMA-NMA. Figure S3. Bar plots on whether transitivity was evaluated as planned among systematic reviews published before and after the PRISMA-NMA that planned transitivity evaluation in the methods section. Figure S4. Bar plots on the conclusions regarding transitivity among systematic reviews published before and after the PRISMA-NMA statement that discussed transitivity. Figure S5. Bar plots on the parameters considered when discussing the implications of transitivity evaluation on the network meta-analysis results among systematic reviews published before and after the PRISMA-NMA statement. Figure S6. Bar plots on the structure of the table of characteristics reported in systematic reviews (plot (a)) and the location in the systematic review report where the table of characteristics was found (plot (b)) among systematic reviews reported in systematic reviews published before and after the PRISMA-NMA statement. Figure S7. Bar plots on whether (and which) PRISMA statement was mentioned in the report among the 60 systematic reviews published in 2016 and those in 2021. [file 12916_2024_3322_MOESM2_ESM.docx]

**Additional file 2**

**Supplementary material for the manuscript entitled 'Low awareness of the transitivity assumption in complex networks of interventions: a systematic survey from 721 network meta-analyses'**

Loukia M. Spineli^1^, Chrysostomos Kalyvas^2^, Juan J. Yepes-Nuñez^3,4^, Andrés Mauricio García Sierra^3,5^, Diana C. Rivera-Pinzón^3^, Svenja E. Seide^6^, Katerina Papadimitropoulou^7^

^1^Midwifery Research and Education Unit, Hannover Medical School, Hannover, Germany

^2^Biostatistics and Research Decision Sciences, MSD Europe Inc., Brussels, Belgium

^3^School of Medicine, Universidad de los Andes, Bogotá, Colombia

^4^Pulmonology Service, Internal Medicine Section, Fundación Santa Fe de Bogotá University Hospital, Bogotá, Colombia

^5^School of Global Health Management and Informatics, University of Central Florida, Orlando, USA

^6^Institute of Medical Biometry, University Hospital Heidelberg, Heidelberg, Germany

^7^Health Economics and Market Access, Amaris Consulting, Lyon, France

**Supplementary figures**

**Figure S1.** Bubble plot on the distribution of the health fields among systematic reviews published before and after the PRISMA-NMA statement.

**Figure S2.** Bar plots on location in the systematic review report where the transitivity notion was found among systematic reviews published before and after the PRISMA-NMA that defined transitivity (plot (a)): 130 systematic reviews published before and 88 systematic reviews published after PRISMA-NMA defined transitivity in their report. Bar plots on location in the systematic review report where conclusions about transitivity were found among systematic reviews published before and after PRISMA-NMA that discussed transitivity (plot (b)): 128 systematic reviews published before and 137 systematic reviews published after PRISMA-NMA stated their conclusion about transitivity *in at least one* location in their report**.** A, introduction; B, methods; C, results; D, discussion; E, supplementary material.

**Figure S3.** Bar plots on whether transitivity was evaluated as planned among systematic reviews published before and after the PRISMA-NMA that planned transitivity evaluation in the methods section: 190 systematic reviews before and 252 systematic reviews after PRISMA-NMA stated in their methods section how transitivity was evaluated.

**Figure S4.** Bar plots on the conclusions regarding transitivity among systematic reviews published before and after the PRISMA-NMA statement that discussed transitivity: 128 systematic reviews published before and 137 systematic reviews published after PRISMA-NMA reported their conclusion for transitivity.

**Figure S5.** Bar plots on the parameters considered when discussing the implications of transitivity evaluation on the network meta-analysis results among systematic reviews published before and after the PRISMA-NMA statement: 105 systematic reviews published before and 94 systematic reviews published after PRISMA-NMA considered *at least one* parameter when discussing the implications of transitivity evaluation.

**Figure S6.** Bar plots on the structure of the table of characteristics reported in systematic reviews published before and after the PRISMA-NMA statement (plot (a)). Dark blue refers to a table of characteristics that facilitates transitivity evaluation regarding the comparison comparability. Light blue refers to a table of characteristics that hinders transitivity evaluation. Bar plots on location in the systematic review report where the table of characteristics was found among systematic reviews published before and after PRISMA-NMA (plot (b)). Three hundred thirty-six systematic reviews published before and 344 systematic reviews published after PRISMA-NMA provided a table of characteristics in their report. A, trial level with characteristics presented for each trial; B, comparison-clustered trial level with characteristics presented for each trial clustered at the corresponding comparison; C, comparison level with characteristics summarised for each comparison; D, intervention level with characteristics summarised for each intervention; E, characteristic level with descriptive statistics reported for each characteristic.

**Figure S7.** Bar plots on whether (and which) PRISMA statement was mentioned in the report among the 60 systematic reviews published in 2016 and those in 2021. Four of the systematic reviews published in 2021 mentioned in their report to have been reported according to the PRISMA-NMA statement had cited an old PRISMA version for pairwise meta-analysis (PRISMA-MA) [37] and two ISPOR reports [24, 38]. MA, pairwise meta-analysis.
